# Coronary Artery Fistula Diagnosed by Echocardiography during NSTEMI: Case Report and Review of Literature

**DOI:** 10.1155/2019/5956806

**Published:** 2019-08-14

**Authors:** Angelo Acitelli, Sabrina Bencivenga, Maria B. Giannico, Chiara Lanzillo, Luciano Maresca, Renata Petroni, Maria Penco, Leonardo Calò, Silvio Romano

**Affiliations:** ^1^Cardiology, Department of Life, Health & Environmental Sciences, University of L'Aquila, Italy; ^2^Division of Cardiology, Policlinico Casilino, Rome, Italy

## Abstract

Coronary artery fistulas are rare abnormal connections between a coronary artery and a cardiac chamber or a major vessel. Often, they are asymptomatic and the diagnosis is accidental. The case we present is the incidental finding of a fistula displayed with echocardiography during acute coronary syndrome (ACS). A 73-year-old man presented in the emergency room for non-ST-elevation ACS. Echocardiogram showed in a parasternal short axis view an abnormal diastolic flow inside the ventricular inferior wall. Angiography and CT confirmed the diagnosis of coronary fistula from the right coronary into the left ventricular cavity. A literature analysis with discussion about coronary fistulas classification and management was also performed.

## 1. Introduction

Coronary artery fistulas are rare abnormal connections between a coronary artery and a cardiac chamber or a major vessel. They are often asymptomatic and the diagnosis is accidental. The case we present is the accidental finding of a fistula displayed with echocardiography during acute coronary syndrome (ACS).

## 2. Case Presentation

**Figure 1:**
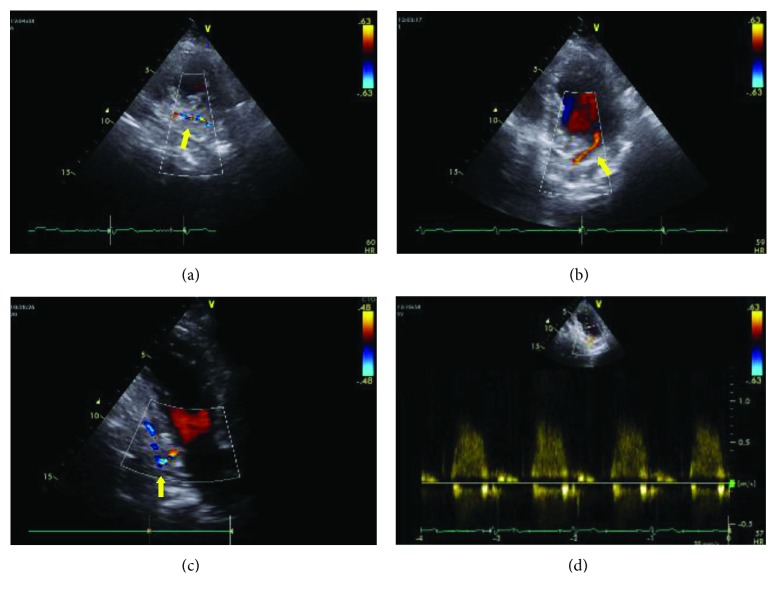
Midventricular (a) and basal (b) short axis view and 2-chamber (c) with color Doppler, showing fistula's course in the left ventricle inferoposterior wall (arrows). (d) PW-Doppler recording fistula flow that is predominantly diastolic.

A 73-year-old man, with a history of hypertension, diabetes mellitus, and hypercholesterolemia, presented in the emergency room because of sudden onset of chest pain at rest. ECG abnormalities associated with an increase of cardiac enzymes (hs troponin 70 pg/ml and CK-MB 8 ng/ml) were suggestive of an ACS (non-ST-elevation myocardial infarction). The patient was admitted to the Coronary Care Unit. Echocardiogram showed left ventricular hypertrophy (septum 14 mm), end-diastolic diameter (53 mm), and mild hypokinesia of the basal segment of the inferior wall with normal systolic function (EF 55%). In the parasternal short axis view, we noticed an abnormal diastolic flow inside the ventricular inferior wall. This flow was directed from the basal segment of the inferior wall into the left ventricular cavity. In the apical two-chamber view, we could follow its entire intramural course, from the apex to the basal portion of the left ventricle, under the mitral valve posterior leaflet, where it was thrown into the ventricular cavity during diastole. Pulsed Doppler sample volume positioned at the level of the flow into the left ventricle confirmed that it was a diastolic flow (Figure 1). The echocardiographic data were suggestive for coronary fistula.

**Figure 2:**
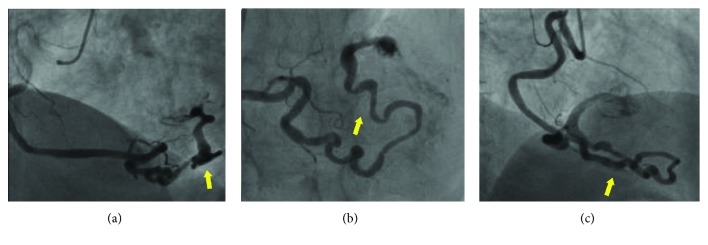
Coronary angiography: right coronary artery stenosis of 50% at the end of the proximal portion. Presence of a tortuous voluminous coronary fistula (arrow).

According to ESC guidelines, the GRACE (Global Registry of Acute Coronary Events) risk score of the patient was 146 and early invasive strategy of myocardial revascularization (within the first 24 hours from admission) was indicated. The patient underwent coronary angiography, which revealed 90% stenosis in the middle portion and 70% stenosis in the distal portion of the left anterior descending artery, a double 70% stenosis in the proximal and the middle portion of the circumflex artery and the right coronary artery, dominant, with stenosis of 50% at the end of the proximal portion. Angiography confirmed the presence of a tortuous voluminous coronary fistula: it originated from the interventricular posterior artery and coursed along the posterior interventricular sulcus from the apex to the base of the left ventricle. The terminal portion of the fistula (approximately two-millimeter vessel diameter) crossed the inferoposterior wall of the left ventricle under the mitral annulus, and with a phasic flow, typically diastolic, it went into the ventricular cavity (Figure 2). The patient underwent percutaneous coronary angioplasty with the placement of two drug-eluting stents in the left anterior descending artery (2.5 × 18 mm in the distal tract and 2.75 × 28 mm in the middle tract) and a single drug-eluting stent (2.75 × 33 mm) in the circumflex artery.

**Figure 3:**
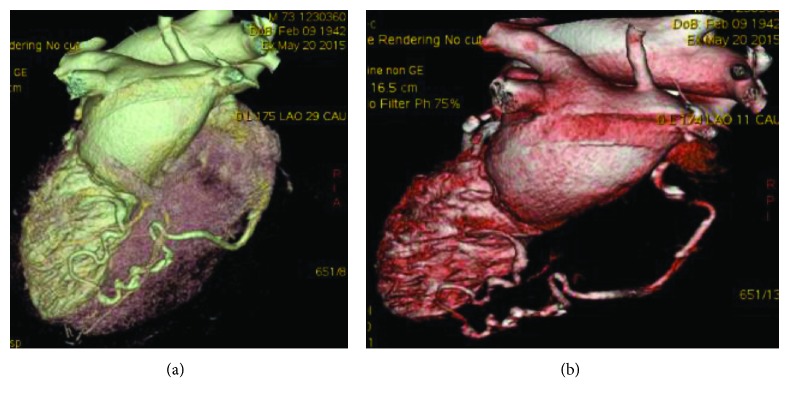
Three-dimensional (3D) reconstruction of the right coronary artery and fistula using computed tomography angiography.

To complete evaluation, cardiac CT was performed to detect coronary fistula anatomy, its relationships with cardiac structures and its course. CT images documented that coronary fistula originated from the distal portion of the right coronary artery, ran into the left ventricular inferior wall, and drained into the left ventricular chamber under the mitral valve, with a final tract with an intramyocardial course (Figure 3). Because the coronary artery fistula did not determine a hemodynamic overload, it was not treated by angioplasty or surgery. At 24-month follow-up, the patient was asymptomatic.

## 3. Review of Literature and Discussion

Coronary fistulas are defined as an abnormal communication between a coronary artery and a cardiac chamber (“coronary-room fistula”), bypassing the capillary bed or any part of the systemic or pulmonary circulation. The first description was by Krause in 1865 and the first surgical treatment was reported by Bjork and Crafoord in 1947 [1]. Fistulas are rare anomalies. They are present in 0.002% of the general population, and they are found in 0.25% of patients undergoing coronary angiography [2]. In an autoptic series of 18950 autopsies, Alexander and Griffith found 54 coronary anomalies (0.3%) [3]. They are often asymptomatic, so their diagnosis is often incidental. Epidemiological data and their incidence may be underestimated in literature. Coronary fistulas may be congenital or acquired. Most of the fistulas are congenital, and their embryological origin appears to be due to the persistence of sinusoidal connections between the lumens of the primitive tubular heart. The acquired forms may be further divided into iatrogenic (during percutaneous coronary intervention, cardiac surgery, myocardial biopsy, and septal myectomy), traumatic, or related to a disease (such as myocardial infarction, Takayasu arteritis, and cardiomyopathies) [4, 5]. Several classifications have been proposed. The first one was by Ogden in 1970, placing coronary fistulas between major coronary anomalies [2]. In 1999, Angelini proposed a new classification of congenital coronary anomalies, identifying anomalies of origin and course, intrinsic coronary anomalies (myocardial bridging, aneurisms > 1.5 mm) and termination anomalies. According to this scheme, fistulas are anomalies of termination [4].

Fistulas are also classified, according to the scheme of Sakakibara et al., into two types: type A, which presents proximal vessel dilation from which originates the fistula, and type B, with the expansion of the entire vessel [6]. Most originates from the right coronary artery and the left anterior descending, less frequently from the circumflex artery. The origin of fistula is rarely bilateral, involving both right and left coronary arteries. In more than 90% of cases, fistulas drain to venous system (in order of frequency: right ventricle, right atrium, pulmonary artery, and coronary sinus), rarely in the left chambers [7] or in the pericardium [8]. Said described the fistulas according to the number of vessels (single channel or multiple channels) and their course (linear or serpentine). Single fistulas are much more frequent than multiple ones [9].

From the pathophysiological point of view, the main problem is shunt entity. It is determined by the size of the fistula and the pressure difference between the coronary artery and the chamber into which the fistula drains. The fistulas that drain into the right-sided chambers (low resistance system) may cause volume overload with hemodynamic impairment, while drainage into the left chambers (high resistance system) leads to a lower overload but may cause an arterial runoff with dilatation of native vessel. In general, small fistulas do not cause symptoms. Larger fistulas can lead to the “steal phenomenon,” which is the reduction of myocardial blood flow distal to the site of the fistula, resulting in myocardial ischemia, more evident in combination with increased demand of oxygen, such as during exercise [10].

Natural history is variable: some close spontaneously, while others persist. It may happen that the coronary artery which originates fistula gradually dilated up to frank aneurysm, while the fistula may be complicated by ulceration of the intima, degeneration of the media, atherosclerotic plaques, calcification, mural thrombus, and rarely rupture [1]. The clinical manifestations increase with age. The most frequent symptoms are dyspnea on exertion, angina, fatigue, palpitations, and paroxysmal nocturnal dyspnea. The gold standard for the detection of coronary fistulas remains coronary angiography. Other imaging techniques such as MRI and CT may provide additional diagnostic elements thanks to 3D reconstructions. Transthoracic and transesophageal echocardiography is useful especially in the evaluation of the hemodynamic effects of the fistula on cardiac chambers [11].

The closure of the fistula is recommended when it is symptomatic, while the treatment in asymptomatic patients remains controversial. Large coronary fistulas should be closed by transcatheter or surgical treatment, regardless of symptoms, while small to moderate size fistulas should be treated only if they cause symptoms [12]. The surgical approach is ligation of epicardial fistula, less frequently intraluminal endarterectomy. Transcatheter closure may be performed with various types of devices (stents, umbrellas, balloons, coils, etc.) but requires favorable anatomy, i.e., not tortuous artery with single fistula and accessibility of the distal portion to closure device [12].

The peculiarity of our case, compared to others reported in the literature [13], is the echocardiographic diagnosis during an acute coronary syndrome. The suspicion of coronary fistula draining in the left ventricle was given by the finding of an abnormal color flow in the ventricular cavity and its route in the wall. Our deductions were then confirmed by coronary angiography and CT. As reported in the literature, fistulas draining into the left ventricle are extremely rare [7]. Echocardiography can give a suspicious diagnosis when there is a hemodynamic impairment, but in our case, it happened during a routine examination by the detection of an abnormal coronary flow in the myocardial wall. The patient's angina was not determined by the fistula but by the presence of atherosclerotic plaques on the left anterior descending and circumflex artery. In fact, the fistula drained into the left ventricle without making any hemodynamic impairment. Therefore, no indication was given for its closure.

## 4. Conclusions

Coronary artery fistulas are described as a direct connection between a coronary artery and one of the cardiac chambers, large vessels, or other vascular structures. They are usually congenital or acquired in rare cases. Most of them are asymptomatic; in fact, small fistulas do not cause any hemodynamic impairment. Some patients, typically with larger fistulas, present symptoms such as fatigue, dyspnea, angina, heart failure, pulmonary hypertension, or infective endocarditis. Often, signs of ventricular overload may be observed by echocardiography. Sometimes, an abnormal flow is observed in the drainage chamber. More rarely, the course in the ventricular wall may be followed with the echocardiogram. The peculiarity of this case is the echocardiographic description of a fistula draining into the left ventricle. In fact, the echocardiographic visualization of a fistula is a rare event. Our patient showed no signs of left ventricular overload, so the fistula was an occasional finding that occurred during ACS. Its closure was not indicated because it did not cause any hemodynamic overload or any symptom, so conservative treatment was preferred.

## Supplementary Materials

Supplementary Materials The supplementary material is only one figure, which could be described as “Admission EKG.”
