# Supplementary figures and images for: Coronary Artery Fistula Diagnosed by Echocardiography during NSTEMI: Case Report and Review of Literature
Source: Case Rep Cardiol. 2019 Aug 14;2019:5956806. doi: 10.1155/2019/5956806 (PMC6710726; doi:10.1155/2019/5956806)

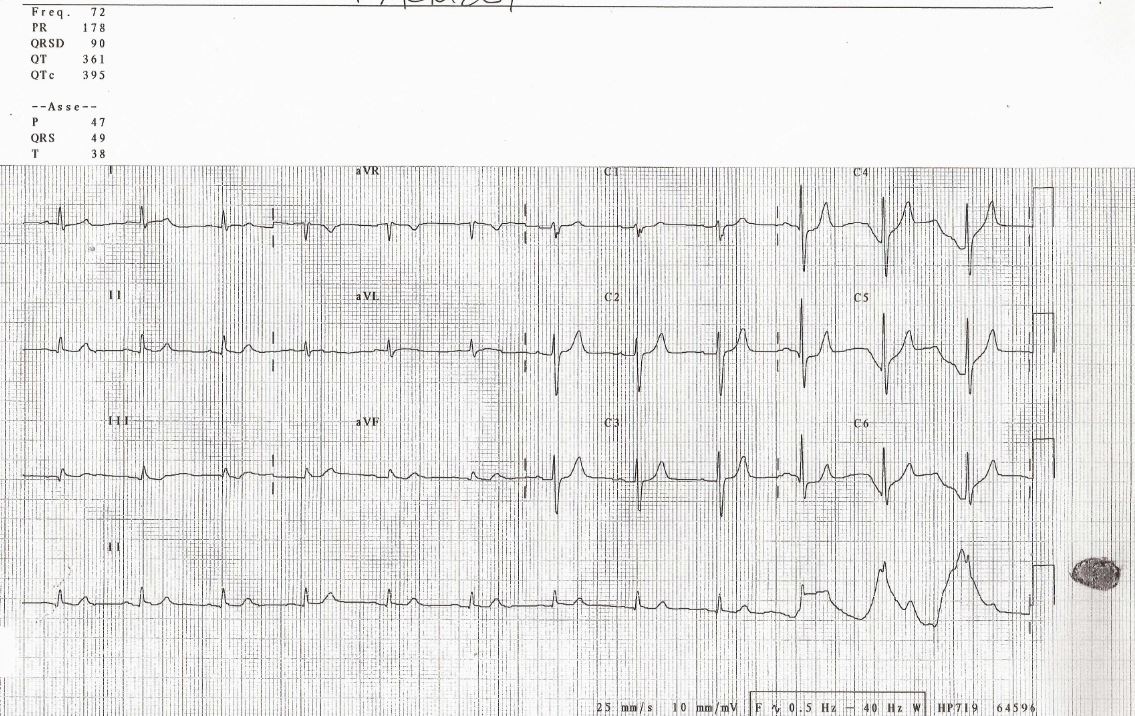

Supplement: Supplementary Materials — The supplementary material is only one figure, which could be described as “Admission EKG.” [file 5956806.f1.jpg]
